# The experience of laser light feedback in back-squat resistance training

**DOI:** 10.3389/fspor.2023.1181371

**Published:** 2023-05-30

**Authors:** N. Stien, V. Andersen, T. E. J. Solstad, A. H. Saeterbakken, G. H. Engelsrud

**Affiliations:** Faculty of Education, Arts, and Sports, Department of Sport, Food, and Natural Sciences, Western Norway University of Applied Sciences, Sogndal, Norway

**Keywords:** visual feedback, phenomenology, embodiment, inter-kinaesthetic, automated, strength, technology, intracorporeal

## Abstract

**Introduction:**

The purpose of this paper is to contribute to the existing literature on performance in resistance training (RT) by addressing how a phenomenological perspective on experiences with inter kinaesthetic affectivity can illuminate experience of practicing RT with non-verbal, visual feedback provided through laser lights attached to the barbell.

**Method:**

The material is created from qualitative interviews and using inter-kinaesthetic affectivity as analytical lenses.

**Results:**

The findings show how participants interpret the feedback in the moment and explain how they adjust their movement in dialogue with the feedback and enable the “uptake” of feedback in their embodied experience. The findings show how the participants developed an awareness of how they can equalize the balance on their feet.

**Discussion:**

We discuss what this means for the understanding of the training process in terms of how practitioners can use the uptake of non-verbal, visual feedback to immediately adjust the quality of their performance by responding kinaesthetically and bodily. The discussion contributes to the question of what kind of role a practitioner's own kinaesthetic and bodily experiences have in the development and organization of RT. Perspectives that include the lived and intersubjective body as a knowledge position are promising for illuminating the whole bodied engagement that is necessary to understand how to perform RT.

## Introduction

Performance in resistance-training (RT) is usually examined by using quantified measurements, and the research interest usually lies in the effects of the RT. To teach and improve the execution of RT exercises, trainers, instructors, or teachers often provide verbal feedback on how the training individual can correct their movements (1). It has been shown that the presence of an instructor can assist in both learning (e.g., provide instructions) and motivation during RT. Personal communication of feedback is strengthened by the instructor's skills in relaying the information, as well as by their experience and ability to individualize the feedback. Importantly, the specific feedback of a trainer is influenced by their experiences and expectations, possibly leading to projecting these preconceptions onto the trainees. Since experiences and expectations are socially constructed, this gives rise to different interpretations between the trainer and the trainee, that can challenge the subjective experiences of both parts in such a relationship (1). A rather novel approach to feedback is the use of non-verbal, technological instructions which can present valuable and use cases and objective feedback in different movements.

Researchers have examined the feasibility of using non-verbal feedback and instructions in correcting bodily movements such as laser lights in RT (2), tactile information in snowboarding (3), and auditive feedback in golf swing (4). These studies concluded that non-verbal feedback (i.e., tactile instructions, sounds, or lights) was potentially useful for correcting and instructing movements. Importantly, these studies were cross-sectional and, therefore, do not inform us about the potential longitudinal effects these feedback methods may have on the efficacy of the training or on the trainers' and participants’ own experiences. Moreover, the feedback method was not compared to traditional, verbal instructions. Still, these studies present novel and interesting ideas for future research into how able participants may be to understand and utilize the non-verbal feedback if the instructor becomes more absent and provides fewer verbal instructions.

Examining the feasibility of using body-lights to inform RT-movements (2) and tactile instructions to correct snowboard technique (3), the authors briefly outline how the participants were instructed on using the feedback and indicates that the participants could utilize the information provided by the feedback independently after only a brief introduction. Interestingly, Spelmezan (3) also interviewed the participants after completing the trials to elucidate their perspective and how they generated meaning of the feedback. The authors concluded that (1) the non-verbal feedback was experienced as useful for the participants, but (2) that non-verbal instructions should not be applied during the first sessions when introducing new movements. Instead, they proposed that the first sessions should include verbal instructions only, followed by a gradually increasing focus on the non-verbal, automated feedback. A period of an instructor introducing the non-verbal feedback method is likely needed for people to be able to independently understand and utilize it (3). This relation between the instructor's use of feedback and the participant's interpretation of it is sparsely described in previous research and warrants further examination. In a recent publication (5), our research group conducted, to our best knowledge, the first intervention study comparing verbal and non-verbal feedback (i.e., visual via laser lights) in developing experience in the back-squat exercise. We followed a similar procedure to what Spelmezan (3) proposed. However, we introduced both methods (i.e., non-verbal and verbal) from the start and gradually decreased the instructor's feedback, thereby allowing the participants to independently use and interpret the non-verbal feedback from the lasers attached to the barbell. The findings indicated that, despite indicative measurements of lifting technique remaining unchanged, the participants increased their maximal strength and self-selected training resistance without an impairment of the technical execution. By interviewing the participants that received non-verbal feedback via the laser lights, we can further examine how it was understood, experienced, and utilized by individuals with no prior experience with either the exercise or the feedback method.

In this article, we will use the concept of inter-kinaesthetic affectivity which we suggest that the phenomena examined in the previous studies can be linked to (2–4). This concept describes how informative and influential the feedback is and how readily the individual understands and allows the stimulus to affect their movement (6). However, it calls upon a further examination since little is known about how inter-kinaesthetic affectivity plays a role in the training individual's uptake and utilization of non-verbal, visual feedback compared to verbal instructions. In the current context, one can imagine that verbal instructions and the understanding of these cues are influenced by individual experiences and interpretations of words and phrases. Hence, it is possible that verbal instructions in RT can be interpreted in a different way than what the instructor intended and that some meaning is changed in the interpretation process between the instructor and the instruction-receiver. To mediate the information-processing during the uptake of data from stimulus, it has been suggested that humans tend to off-load a portion of the cognitive work onto the environment and collect information on a need-to-know basis to be replaced with kinaesthetic awareness (7).

Non-verbal feedback in the context of RT can originate from the trainee vi lights attached to the person or the equipment (2, 5). This approach may create a form of inter-affective relationship between the trainee and the perceived stimuli from the non-verbal feedback where they interact with- and move each other in a circular relationship (8). The expression of movement instantly prompts a related impression that is tailored and synchronized to the individual person and their movement pattern. To date, the existing literature provides limited knowledge about the personal experience of interpreting feedback while conducting RT, and current research has relied mainly on quantitative methods. Consequently, there is limited insight regarding the experiences people have in receiving and interpreting feedback. However, there is a growing interest for the lived experiences and personal interpretations of feedback (9). Therefore, we are interested in whether this potential inter-affectivity introduces a bodily knowledge that is readily available for the training individual. Moreover, we ask the question whether such knowledge can be understood and taken up effectively since the environment is moved in a perfect interrelation with the individual expression of movement. Since the personal experience of those who undertake RT is of importance for whether they continue the RT in the long run (10), it is desirable to understand how people interpret and experience feedback when they participate in RT. We designed an interview study exploring (I) how young, women without RT experience interpret the non-verbal feedback during RT and (II) how the women's interpretations of non-verbal, visual feedback may allow for new insights about the approaches available for RT instruction. Our questions aim to explore the structure of the bodily awareness that should be elucidated to understand how progress and satisfaction in the practice occur.

## Materials and methods

### Design

We designed a qualitative interview project to address the research questions and used semi-structured interviews to explore the informants’ lived experiences of receiving non-verbal feedback from the laser lights during RT. The interviews were guided by a phenomenological paradigm, informed by perspectives on movement experience from Fuchs and Koch (8), Sheets-Johnstone (11), Behnke (6, 12), and Zahavi (13). We used the research literature on embodiment and lived experience as a backdrop to apply an approach to qualitative research that allowed us to engage proactively and exploratively with the informants. We also took inspiration from Zahavi (13) who argues that “qualitative researchers should rather strive to let their own research be informed by central phenomenological concepts such as lifeworld, intentionality, empathy, horizon, historicity, and the lived body”. Zahavi (13) inspired us to be aware that conducting phenomenologically informed qualitative research is not merely a question of being open-minded and interested in first-person experiences. It is also very much about adopting and employing a comprehensive theoretical framework concerning the subject's relation to themselves, to the world, and to others (13).

In a phenomenological approach it is central that researchers are open and aware of the phenomena in the study. This indicates that to investigate experiences of performance in RT, it is necessary to take into consideration that the training experiences might be difficult to articulate, and therefore give time and space for “letting the informants be” and trust their own words (14) Indeed, most bodily habits and the perception of one's own body might go unnoticed by the consciousness in daily life and even in training practises. Hence, we designed the project while being aware that getting a substantial material would rest on our way of integrating openness and creating the potential to describe and analyse the phenomenon of RT in a novel way (15). It means to search for substantial descriptions from the informants’ meaning generation in their own words (16). To allow us to reach a deep insight into the experiences of the participants, we sought to learn about their interactions with- and understanding of the feedback and generate meaningful knowledge by analysing their experiences using concepts such as inter-kinaesthetic affectivity and intersubjectivity.

### Selection and recruitment

A purposive sampling technique was used to recruit informants who could illuminate the research question (17). Five females with no prior systematic RT experience in the last eighteen months were recruited from larger group of ten participants who were taking part in a quantitative study examining the feasibility of using visual, non-verbal feedback in developing the back-squat technique (5). The study compared the changes in strength and technical execution of the back squat following five weeks (10 training sessions) of training while receiving either verbal or visual feedback. Throughout the intervention, each session included three sets of ten repetitions with a self-selected external load that corresponded to approximately two repetitions in reserve and an eight out of ten rating of perceived exertion (0 indicating no exertion at all and 10 being maximal exertion). The verbal feedback was standardized by only using a selection of four ques that were developed in cooperation with a group of highly experienced personal trainers. The visual feedback group received information about their spatial positioning (i.e., frontal, sagittal, and transverse planes) from laser lights projected on a reflective whiteboard with lines indicating how the lights should travel during each lift. Briefly described, the laser lights were attached to the barbell and moved in concert with the participants' movements, making minor deviations from a straight line immediately visible. The participants were novices in the exercise, but displayed acceptable technique when the external load was within a self-selected, manageable range. Hence, the role of the feedback was to correct the technique rather than introducing the participants to the exercise. During the first two training sessions, the instructor was present and assisted the participants in generating an understanding of how to interpret the laser lights' feedback. In the last eight sessions, the instructor only provided motivational support and was available for discussion and reflection about the training. The informants gave their written informed consent as well as verbal assent before the interviews began. We reminded them before the interviews about their right to withdraw from the study at any time without any negative consequences, and about how their anonymity would be maintained (18). Furthermore, we made sure to clarify our roles in the interview context as researchers and highlighted the fact that we were interested in hearing about the informants' lived experiences since they were the insider experts in this context (19). The research procedures were processed by the Norwegian Centre for Research Data (reference 440571) and ethical approval was granted by the university's local ethics committee (reference 21/08477-3).

### Material creation

We conducted a total of five semi-structured research interviews in October 2021. Interviews took place immediately after the participants had completed their fifth out of ten prescribed training sessions. The last author (GHE) conducted three interviews and the first author (NS) conducted two interviews due to logistical reasons. Importantly, both authors were present in the first interviews to ensure that the setting was as similar as possible on the final three interviews. However, the authors also remained mindful of the importance of allowing the natural intersubjectivity that arose between the researcher and the informant in the specific times and places to take place, and not compromise the flow of the dialogues by attempting to make the interviews more similar. Each interview started with the researcher verbally repeating information about the project, the research question, expected duration of the interview, as well as how we would maintain the participants' anonymity. The semi-structured interview guide comprised the two following open-ended questions: (1) “Could you tell us about your experiences from the training with the non-verbal feedback?” and (2) “How would you describe your own change or improvement in the exercise following the training?”. As already stated, we used a phenomenological and reflective approach to the interviews, which opened the opportunity to ask follow-up questions about the themes that the informants themselves chose to bring up in their answers (20). This happened when they talked about the meanings of how the body responded to the laser light. We became aware of a way of verbalizing experience and discovered that asking follow-up questions gave insight into the informants' way of expressing their experiences (21). As Rapley (22) clearly express, interviews are also highly dependent on- and emerge from the specific local interactional context which is produced in and through the conversation. By recognising and asking follow-up questions we obtained substantial descriptions, which we consider the argument for choosing interviews (20). It means that interview is not only about asking, but as already mentioned, to be remain aware that the phenomenological approach seeks to uncover the intersubjectively accessible structures that may be shared between interlocutors (23). Hence, the accounts of the training were co-produced in the encounter between the researchers and the informants. In addition, Depraz et al. (24) explicitly accounts for the role of the researcher's sensitivity and are concerned with the researcher's responsibility for creating space for verbalization of experiences, to make available what is not spontaneously expressed. Each interview lasted between 15 and 30 min. The interviews were recorded and transcribed verbatim using Microsoft Word (Microsoft, Redmond, WA).

Work on the analyses started informally immediately after the interviews. We were particularly aware of how the different participants accounted for their version of the training experience and that the material was produced in a local context in the actual training space (25). The voices of the informants and the dialogical approach to the interviews were the most important guidelines and we identified those parts of the informants' expressions that could shed light on the research questions (26, 27). The material from the interviews provided an opportunity to explore the lived experiences of the informants that they were able to articulate. We began by reading the transcripts and listening to the tapes to get a sense of the whole material, which is a general recommendation in qualitative research (28). After getting a sense of the whole material we used the method's whole-part-whole procedure to further work with the material and started to re-tell what the informants had told us (16). Our strategy for the further analyses was to listen to the recordings and read the transcripts to grasp the meanings of how the participants expressed how they created meaning from training and interacting with the laser lights' feedback. By suspending our reading of the material through the concept of bodily inter-affectivity, we started to discover how these phenomena functioned as underlying premises for how the process seemed to be progressing according to the participants. We first identified some of the experiences that the women had in common, albeit expressed using slightly different phrasing. Next, we used their expression to identify themes and experiences that we could re-formulate and thereby elucidate the most (16). By staying close to the expressions that appeared in the material and avoid “intrusive and overly imposing interpretations”, we used the informants' own words and expressions. With the support of the chosen perspective, we were able to elaborate on the experiences. Rather than using a rigid and predefined process of identifying the themes that occurred most frequently in the material, we allowed ourselves time to get as familiar as possible with the transcribed interviews to get a clear sense of what was perceived as important and meaningful by the informants. Moreover, our experiences from the interviews (e.g., body language and when the informants appeared enthusiastic) also helped us in identifying and creating themes that resonated with the lived experiences of the informants. Our discoveries in the material helped us use and activate the theoretical framework and we conducted a circular process of familiarizing ourselves with the material and exploring relevant concepts and literature throughout the work with re-formulating the information from the interviews (29). Finally, we continued with some phenomena where the phenomenological framework was used to create the headlines that we discovered in the interviews.

## Results

The following section will present the primary recurring themes that emerged from the interviews.

### “I did not know how I moved”

The informants seem to have realized during the training that they lacked an innate awareness of how their bodies moved. In other words, they explain how they were previously unable to feel the positioning and movement of their body parts, and phenomenologically speaking, they were in the “natural attitude”, like one who stated that “… before, I didn’t know that I loaded the left foot so much, I had no idea that I did that” and continued to explain how she became “very surprised that I could be so little aware of it”. Another informant, when asked if she could feel postural changes in the body during the training, told us “No, usually not” and elaborated that she only might have felt any postural change after continuing with poor lifting technique for a while. Several statements from the informants indicated that albeit challenging to *feel* their body position, receiving the information from the laser light has helped them recognize some bodily feelings and become able to connect the feelings to specific positions or movements. In general, the informants expressed a realization that indicates that when they started to perceive the feedback within their own bodily receptivity, the back-squat training gave them new meaningful information. They told us that they became aware of their bodily movement patterns that they reflected on not being aware of by training without feedback or using a mirror. These experiences became deepened in the theme which we will present in the next section.

### “It was difficult in the beginning—until I learned to interpret the laser light”

The second theme that emerged from the interview material illuminate the participants' descriptions of how they felt like they needed to learn how to utilize the non-verbal feedback before their bodies fully grasped the meaningful communication with the laser light. One of the informants explained how it was difficult to “see what was right and wrong” based on the feedback. She then went on to tell us how she needed the instructions from the research leader in the first phase of the training to learn what the different indicators from the laser lights meant in relation to the movement. When she learnt how she could interpret the feedback, and that she could then go on to practice more independently from the research leader's guidance. The same reflections arise from other interviews, with one who explained that “it is difficult to learn [the back squat technique] based on the laser if you don’t have knowledge about the laser”. The same informant said that “there must be both for me to learn something”, referencing the non-verbal feedback and the exercise itself. In other words, the early phase of the training where the research leader was present and introduced them to both the feedback and to the exercise appears important and meaningful for the informants. These and other statements from the informants also highlight the fact that there are two elements that need to be learned: (1) how to perform a back squat and (2) how to use the non-verbal feedback in a meaningful way in their execution of the exercise.

### The laser light and the body in inter-affective communication

The informants have also learnt how to create balance on their feet and based on the feedback. Many of the informants described the uptake of the feedback as an automatic process where the laser light communicates directly with the body. One expressed her experience in the following way: “it's not the foot that controls the light, but it's the light that controls the foot”. The same informant went on to describe a process that had occurred where she had become able to control the laser light by immediately correcting her movement if the feedback informed her that she was off balance. Another explained that she “… can see much more clearly when the technique becomes poor”. The informants appear to have learnt the laser's way of informing them about their movements and become able to respond to its feedback rapidly and in a way that results in a correction of the errors and deviations in the movements. Once the participants learned how the laser functions, they began an even deeper communication between the body and laser as an inter-affective circle. Of note, one informant told us that “… the heavier the load becomes, the easier it is to feel which foot I place the most weight on,” and continued by explaining that “… the laser makes more sense the heavier the load becomes”. Later, she described that her focus was increased along with the loading.

### The laser light vs. other forms of feedback

As noted above, the informants described an increased understanding of the non-verbal feedback and ability to respond to it in a rapid and appropriate way. Some of the informants further compared their new experience with the laser lights' feedback to their previous experiences with receiving verbal feedback from a trainer or instructor. One stated that “it's much easier to read the lights” compared to adhering to verbal instructions. She explained that verbal feedback can be abstract and difficult to transform from listening to the voices to altering their own movements. Verbal feedback can be misunderstood and may not always correspond to how she experienced the movement. Another informant compared the laser feedback to using a mirror and said that “The laser is helpful compared to using a mirror, where you only see it when you're doing it *very* wrong!”. She explained that the laser feedback provided a clearer and more evident picture of her specific movements compared to seeing her whole body in a mirror. The experiences indicate that it is difficult to understand the meaning of kinaesthetic responses in the body. Moreover, it appears that visual information in a mirror or verbal instructions do not provide immediate practical use for correcting one's own movement. Based on how the informants told us about their experiences, the non-verbal nature of the laser feedback appears to have been experienced as useful in a sense that only meaningful and relatable information needed to be harvested from it and that it was understood directly and immediately in the body.

### Perceived safety via the feedback and the research leader

All five informants spoke about and were aware that the research leader played a role in their RT progression. Knowledge and understanding are shaped in a liminal space and the participants need “the other” to understand the laser (the researcher with his knowledge and guidance). They all highlighted that it is important how the researcher spoke to them and that the training took place in a welcoming atmosphere, which they point to as a precondition for their experience with improving their technique. They pointed to a new acquisition of knowledge concerning the “right way to do a back squat”. When they felt the new embodied understanding, they expressed “ownership” and were able to put this knowledge into use, and as one stated: “If I’m ever going to have to do squats, I’ve learned how to do them a little more correctly”. Both the researcher and the laser light together represented safety for one of the women who expressed that “Yes, I think it has been a form of safety to have the lights saying […] something about how my technique is”. Referencing a previous encounter with the back-squat exercise, she explained that “when I was practicing back-squat and was about to add more weight, I was thinking “ok, now I'm adding weight and I know that my technique becomes worse”, but with the lights you sort of get a clearer answer to whether, well, if you're doing it right”. She added “…probably a combination of that (the lights), and that you (directly to the researcher) are there and provides some additional comments. And that I can ask you (directly to the researcher)”. The informant described that in order to benefit from the actual training session, she needed to be familiar with the context, and she underlined that the knowledge of the researcher played an important part.

### Learning with the light—learning without the light

The last major theme that was identified comprised the informants' reflections about how they thought training the back squat without the non-verbal feedback would be for them. One of the women said that “I’m excited to see how it is without the lights when this is over. If the body has actually taken [the feedback] in and can work without it. Or if it's the lights that control it (the movement)”. The same informant went on to recount a session during the intervention where she tried not looking at the lights for one set in order to “feel a little bit” how performing the lifts would be without the feedback. She explained how her experimentation with looking and not looking and the laser lights made her somewhat unstable, and she recalled the research leader's instruction on the specific execution. When asked about how she thought she would experience training without the laser feedback in the future, she told us that “… I think that I would miss it, yes—to actually get an answer from the lights”. These statements raise questions about the position the women give the non-verbal feedback in facilitating improvement in the exercise, as well as the kinaesthetic awareness in RT that women may have acquired or if they may feel overly dependent on the constant feedback of the laser.

## Discussion

In this study we sought to explore the experience of laser light feedback in RT. Feedback is information communicated to the training subject that is intended to modify his or her thinking and/or movements to facilitate development or improve execution. The discussion is centred around the primary themes that were identified in the interview material. It has been suggested that feedback has no intrinsic value (9). Rather, it is the use and uptake of it that count when learning. In the following section, we discuss the findings considering the role of feedback and the meaning the informants created from experiencing the laser feedback.

It is important to note that laser lights as a form of non-verbal feedback is unlikely to provide useful information for individuals that do not have knowledge of how to utilize the information. In our previously published training study (5), we implemented a brief introductory period where the non-verbal feedback was combined with verbal instruction about how to interpret the information from the laser lights. Based on the statements from the informants, this period was perceived as valuable and necessary to be able to independently understand and utilize the non-verbal feedback in the following weeks. Of note, none of the informants reported difficulties in utilizing the non-verbal feedback after being introduced to it and having practiced using it for some sessions. These findings are not surprising but highlight the importance and usefulness of including a familiarization period in the start of training where basic information about the exercise and the feedback is given.

Several of the informants indicated that the laser feedback gave them information about their movements they were not previously aware of. Moreover, one stated that she was not confident that she was able to independently sense postural changes during RT. Learning about feedback in RT with the laser light also seems to raise the participants' awareness of the qualities of their movement. To be aware of how one's body is moving is likely crucial for allowing appropriate correction of movements and body positioning (6, 11, 15, 30). Importantly, the informants explained that being visually informed by the laser about their movements helped them in becoming more aware of how their bodies were positioned in space and towards the ground. Specifically, they reported an increased perceived ability to *feel* how they moved, which is crucial for the ability to navigate meaningfully within one's surroundings (31). Following Behnke (6, 12), it is likely that the kinaesthetic sensations, or feelings, were present before the training intervention, and the increased awareness might be a result of the laser feedback assisting in connecting these sensations to specific movements and positions. As one informant expressed this mechanism, she felt that she had to “… see it in order to feel it”. It is also important to note that the informants' movements provided a feed-forward to the laser lights, which highlights the circularity of moving and being moved as an inter-affective situation (8). Training with the lasers may occur as described by Sheets-Johnstone (11) as movement in concert or in ways that are harmonious. Related to some informants' previous experiences, they told us about how they did not know how they moved. Going from not being aware of their own movements to becoming familiar with how their bodies move in space represents a considerable change for the included informants.

Another interesting perspective that was emphasized by the informants was the experience that they related to the laser feedback in a seemingly unaware manner following the familiarization period. As one stated, the laser communicates directly with the body. Rather, the awareness seems to become apparent for the participants after the movement is completed, indicating a retrospective sense-making (32) due to the laser not “making sense” before it is connected to a kinaesthetic sensation. In a way, the communication between the laser and the body cannot be verbalised but must be experienced. Yet, the participants described that the feedback caused a change in the movement that they became aware of, which helped them further adjusting their movements. This finding speaks for the idea of inter-kinaesthetic affectivity playing a central role in RT movements. This is further supported by the phrasing many of the informants used when describing how they harvested the information the laser gave them, and how they were able to use and interpret the information to their advantage. They stated that they could respond to what the laser “instructed” them to do based on its' relation to a feeling rather than an explicit instruction. In many ways, these accounts correspond well to previous descriptions of embodied cognition (33, 34). When a person is situated in this specific context and can visually sense the environment providing affordances, they can be directly influenced by the environment and adjust their actions with effortless information-processing (35). In this manner, the sensorimotor functions are “body-based” (34) and the central understanding of the information provided by the laser is only realized after it has been interpreted by the body.

The informants' unawareness of their own movements in combination with their explanations of the feedback as having an effortless connection with the body may indicate that the visual, non-verbal feedback can be favourable over traditional, verbal feedback for this specific population. As previously described, feedback in RT is traditionally provided verbally by a trainer or instructor during or after the movement. Some of the informants had previous experience with receiving verbal feedback in RT from a trainer. When reflecting on the comparison between the two methods, one expressed that she had experienced verbal ques from an instructor being interpreted in a different way by her than what the instructor had intended. She highlighted that words and phrases can have different meanings for different persons, and that the externally observed movement may not always reflect her lived experience. Trainees must likely be somewhat aware of their own movements and body positions to understand verbal ques from an instructor (1). Conversely, the informants expressed that the laser provides “a direct indicator of what I must work on” that is directly taken up in the body without having to be interpreted or translated from words to action. Rather than being created by one person interpreting the movement of another and communicated back verbally, the feedback from the laser is both created and received by the same body. This likely presents a constant circularity of feedback and feed forward between the training body and the laser lights. Therefore, the information from the laser is both individually tailored and open to an interpretation that has value and can be understood by the receiver. This *open representation* (36) means that the laser alone does not suggest a meaning or give explicit, instructional data. Hence, one can argue that visual, non-verbal feedback says more than a thousand words, but only the ones that can be interpreted in a meaningful way by the trainee are harvested and utilized. Since the informants were originally unaware of how they moved, a feedback method that seems to bypass the awareness and directly communicate with the way they move may be more suitable for this population since it does not require an explicit understanding of one's own movements.

One informant explained to us that she perceived her own focus to be elevated when she lifted heavier compared to lighter loads, such as during warm-up or in the earliest sessions of the training intervention. It is possible that the stimuli from the non-verbal feedback and from her own body are perceived as stronger or clearer following an increase in the training load. This phenomenon might be a understood through the concept of inter-kinaesthetic affectivity (6) in the sense that both the strength of the external stimuli, as well as the degree of bodily awareness by the individual are elevated when the relative loading is increased. Moreover, this statement corresponds to the findings by Spelmezan (3) who concluded that the perceived level of how helpful the non-verbal feedback was may be related to the relative difficulty of the task. Specifically, the researcher reported that very easy tasks made the participants perceive the feedback as disturbing, whereas very challenging tasks made it difficult to maintain a focus on both the task and the feedback. In our intervention study (5), the training load was self-selected in cooperation with the researcher, meaning that the difficulty of the task was challenging, but not too challenging for the participants. Together, the findings from this study in the light of Spelmezan's (3) reports could indicate that non-verbal feedback in RT is feasible and perceived as useful when the loading is relatively high, but within a somewhat comfortable range.

It appears that the time from feedback is given until an adjustment is made can differ between the two feedback methods. While the laser provides constant and immediate information about the movement, verbal feedback is often given after a movement is completed. Our interpretation of this is that recalling the feeling of a movement makes it challenging to associate it with the verbal feedback in hindsight. However, further research is needed to confirm how these experiences can be understood. One reflection is that verbal feedback also can be given during the movement. However, from the perspective of the training subject it would require a longer process of interpretation and require more information-processing compared to the laser lights' feedback. Indeed, one of our informants described the bodily response to the laser feedback as “automatic” in that the laser controls and is in contact with the body directly. One potential benefit from the immediate adjustment following the laser feedback is that one single attempt can provide the trainee with the answer to how a correct execution should feel (if they are able to correct accordingly in the same attempt). In comparison, verbal feedback may promote a more trial-and-error based approach which might not be perceived as useful until the trainer makes the trainee aware of the intention. This can entail a series of several unfavourable executions before the desired movement pattern is obtained, which furthermore can create a frustrating space for both the giver and receiver of the instructions.

It is important to note that our intervention study (5) found no improvement in lifting technique as subjectively rated by three RT experts. However, the participants that received visual, non-verbal feedback improved strength and their self-selected training resistance throughout the intervention without impairing lifting technique. Moreover, the participants only received verbal ques during the first sessions and trained independently from the instructor in most of their sessions. This could indicate that, although not identified by the measurement methods, some development did occur that allowed the participants to lift more weight with improved independence and self-confidence following familiarization with the exercise and the feedback method. The improved perceived safety that the informants describe likely mediated their increase in self-selected training resistance. However, it appears that they understood the laser feedback more as a tool for helping them to avoid errors than for improving their current technique. Further examination of this phenomenon is required to reach a deeper understanding of how the laser feedback might lead to both improved self-confidence and performance in RT.

It becomes apparent that the informants view the laser lighs' feedback as useful and even more helpful than verbal feedback from an instructor. However, it is important to highlight that they are aware that the feedback does not exist in a vacuum, but as a part of the social setting in the RT context. To create meaning from the non-verbal feedback, they must have some knowledge of both the exercise and the feedback itself, as well as to trust their own bodies and movements. For example, one mentioned that «… it's difficult to learn it (the back-squat exercise) from the laser if you don't have knowledge about the laser. I feel like there must be both for me to learn something”. With “both” she refers to the laser feedback and the researcher who provided information about how to understand the feedback the laser provides. The statements from another woman about how the presence of both the lights and the researcher were necessary for the feedback to have meaning for her highlights that this form of feedback has no intrinsic value unless it has been explained for the participants before they start using it. It also highlights the importance of the presence of an experienced instructor that the participants trust in giving them sufficient and correct information about the lasers’ use case. Given this initial presence of an experienced instructor, one might assume that the participants' bodies were prepared for the kinaesthetic responses between them and the laser. In other words, the training atmosphere reduced possible stress and perceived pressure to perform the exercise well from the beginning of the training. In contrast to the recommendations by Spelmezan (3), our findings can be interpreted as an argument for an early introduction of automated feedback in the practice. Since the non-verbal feedback might be more open for interpretation, it could allow beginners to explore and develop their own definitions of optimal movements which can later be fine-tuned by the assistance of a trainer.

One intriguing theme that emerged when analysing the informants' statements is that they sometimes describe the development they experienced during the training as relayed to them through the laser feedback and not directly through doing the exercise. It is possible that the lights helped them become aware of how their own kinaesthetic sensations were related to the execution of the training only after first becoming aware of the lights. This further supports our speculation that an exploratory introduction to a new movement might promote bodily awareness. This process could produce an important foundation for further practice if the trainees can have a broader repertoire of sensations that they can assign verbal cues to. Again, their frequent usage of the word “automatic” in their descriptions of how they were moved by the laser is noteworthy. This could indicate that when the laser lights communicated directly and non-verbally with the body, the perceived learning that the informants describe reflected their increased understanding of the feedback rather than of the exercise. Indeed, one informant reflects on this matter and asks herself if “… the body has actually taken it in and can work with it, or if it's the lights that control it (the movement)”. When asked to elaborate, she concluded that “I would miss the lights” if she were to train without them and specifies that she referred to the lights giving “answers” to whether she is performing the exercise correctly. These observations indicate that a dependency on the feedback develops after a period of training with the laser lights constantly providing information about the movement. The participants' accounts of their ability to eventually control the lights (feed forward) and comprehend their kinaesthetic sensations regarding their body posture suggest that their movement was gradually transformed into a product of their own volition, rather than a consequence of the feedback they received. Unfortunately, our current data is not able to indicate how this may manifest over time, but this should be explored in future research.

Traditionally, there has been a division between inner and outer stimuli for influencing the movement patterns of training individuals. For example, external stimulus is often provided as verbal instructions or encouragement (37), whereas intrinsic motivation or sensations of contact with the body and movement can be regarded as internal stimuli. To take up external, verbal stimuli, the training individual must process the information that is given to them and translate it into a mode of communication that the body understands and can respond to with movement. Conversely, internal stimuli may be lived and experienced rather than heard and interpreted. By implementing non-verbal, visual feedback, we theorize that the line between the internal and external is blurred, and even erased as the intracorporeal space between the training individual and the laser light is shared and created in unison (38). Indeed, the visual lights are external from the trainee's body, but at the same time they originate directly from them and may be experienced like an extension of their bodies that they can visually observe and take up information from. As one of the informants mentioned, the laser light feedback is comparable to a mirror, but provides clearer indications of right and wrong movements or deviations from the desired movement pattern. Furthermore, the uptake seems to happen automatically and directly, without them having to process the information through thinking *about* the movement. As one of the informants put it “But I never think that, when I see that one (laser light) goes to the side, it's because I have too much weight on one foot. It's more like I don’t know which foot, but I know that I must have it equal on both”. In this statement she describes the visual uptake of the feedback and how she does not need to understand it to know what she needs to do. The experience she described could be exclusive to this specific method of feedback because it is (1) non-verbal, (2) provided directly and immediately, and (3) tailored specifically to the person's movement (by the person's own movement). Albeit not investigated in this context, it could be of interest for future research to explore in what ways the use of visual feedback can assist trainers and trainees in developing a language that takes bodily experiences as reference points that both the trainer's verbal instructions and the trainee's sensations can be linked to. The phenomenological framework can prove useful in future research into this topic.

## Conclusions

In this study, we sought to explore how non-verbal, visual feedback provided by laser lights was experienced and interpreted by inexperienced females undertaking back-squat RT. Following our analyses and interpretations, the interview data suggests that the informants perceived the laser feedback both as a useful tool for learning and maintaining qualitatively aligned technique during the training. Moreover, the informants expressed that they had experienced an improved awareness of their own bodies in the context of RT and feel that they can be more in contact and in control of how they move. They explained the progression in their movements as originating in the visual information which they are, over time, able to relate to kinaesthetic sensations informing them about their movements and bodily positions. Following a period of training and familiarization, they report an increased independence and ability to correct movements based on a combination of the feedback and their bodily sensations. Our previously published training study (5) indicated that increased loading could be used without a depreciation of lifting technique or movement quality when the laser feedback was used. To our best knowledge, however, it is still unknown how training with visual, non-verbal feedback affects long-term development and if it produces long-lasting changes in movement patterns. Trainers and researchers can consider using this form of feedback as it seems to be perceived as useful for the participants. Importantly, a familiarization period together with a trainer or instructor seems necessary to introduce both the exercise and how to utilize the information from the feedback. Further research is needed to identify how much emphasis there should be on either the verbal or the non-verbal feedback at different times in the progression of the practice. Hopefully, this paper can assist in the progress of generating adequate concepts and theoretical frameworks to be aware of when developing and understanding RT programs.

## Data Availability

The raw data supporting the conclusion of this article will be made available by the authors upon reasonable request, without undue reservation.
